# Montreal Cognitive Assessment of cognitive dysfunction after basal ganglia stroke

**DOI:** 10.1007/s13760-022-01967-4

**Published:** 2022-05-28

**Authors:** Baoye Ye, Dingqun Wei, Lin Pan

**Affiliations:** Department of Rehabilitation, The Second Affiliated Hospital of Fujian University of Traditional Chinese Medicine, Fuzhou, Fujian China

**Keywords:** Basal ganglia stroke, MoCA score, Cognitive impairment, Clinicodemographic characteristics, MoCA dimensions

## Abstract

**Objective:**

The Montreal Cognitive Assessment (MoCA) was used to evaluate cognitive dysfunction after basal ganglia stroke, and factors affecting total MoCA score were examined.

**Methods:**

Data were retrospectively analyzed for 30 patients with basal ganglia intracerebral hemorrhage or basal ganglia cerebral infarction, who were admitted to The Second Affiliated Hospital of Fujian Traditional Medical University (Fujian, China) from January 2017 to March 2020. Cognitive impairment was assessed using the MoCA, and potential correlations were explored between clinicodemographic characteristics (sex, age, stroke location and etiology) and MoCA dimensions or total MoCA score.

**Results:**

Univariate linear regression showed that the total MoCA score was significantly associated with sex, age, executive function, naming, attention, abstract generalization ability, memory ability, and visuospatial orientation. However, multivariate linear regression identified only executive function, naming, attention, memory ability, and visuospatial orientation as significantly associated with the total MoCA score.

**Conclusions:**

We showed that the MoCA test can be used for patients with basal ganglia stroke. The total MoCA score of basal ganglia stroke was significantly associated with impairments in executive function, naming, attention, memory ability, and visuospatial orientation.

## Introduction

Stroke is currently the second leading cause of death and the primary cause of disability worldwide [1, 2] leading to somatic motor dysfunction and cognitive decline [3–5]. Cognitive dysfunction after stroke severely affects recovery and quality of life, while it increases the financial burden on families and medical costs to society.

Basal ganglia stroke is a common type of cerebral infarction, while basal ganglia intracerebral hemorrhage is the most common type of intracerebral hemorrhage. Specifically, contralateral hemiplegia, hemidysesthesia, and hemianopia can occur in the basal ganglia, which consist of an inner capsule surrounded by white matter. Subcortical aphasia, in contrast, can occur in dominant hemispheric lesions.

The Montreal Cognitive Assessment (MoCA) is a cognitive screening test used to detect mild cognitive impairment and Alzheimer’s disease, and it is considered suitable for stroke patients [6]. In fact, the MoCA allows more detailed evaluation of cognitive function than the Mini-Mental State Examination and shows greater sensitivity for detecting cognitive dysfunction after stroke [7, 8]. Recent studies have also shown that MoCA screening for cognitive impairment after stroke is simple and effective [9] and can predict functional recovery after 3 months [10].

However, the efficacy of MoCA in assessing cognitive dysfunction specifically after basal ganglia stroke is still unclear. Therefore, in this study, we applied the MoCA to evaluate cognitive dysfunction after basal ganglia stroke and explored the potential association of MoCA subscores in different cognitive domains with the patients’ demographic and clinical characteristics.

## Materials and methods

### Study population

This retrospective study included patients with basal ganglia intracerebral hemorrhage or basal ganglia cerebral infarction who were admitted to The Second Affiliated Hospital of Fujian Traditional Medical University (Fujian, China) between January 2017 and March 2020 and were older than 20 years, were diagnosed with basal ganglia cerebral infarction or cerebral hemorrhage by magnetic resonance imaging, and had the disease longer than 14 days but shorter than 3 months (recovery stage). Patients were excluded from the study if they had cognitive impairment (e.g. Alzheimer’s disease, Louis body dementia) before stroke onset; if they had cognitive impairment due to other causes, such as cerebral trauma, cerebral infarction, or hemorrhage besides basal ganglia; or if they were unwilling or unable to complete the MoCA, such as because of a severe mental disorder.

The study was approved by the institutional ethics committee (ethics review number 2017- KL017-02), and all patients signed written informed consent prior to enrollment.

### Observational indicators

Once patients entered the recovery phase, the Beijing version of the MoCA was used to assess their cognitive function [11], and the assessments were evaluated by a physician with expertise in MoCA evaluation. The MoCA, for which the total score can be a maximum of 30 points, assesses seven cognitive domains: visuospatial orientation, executive function, naming, attention, language function, abstract generalization ability, memory ability, and orientation ability. A total MoCA score below 26 points was considered to indicate cognitive impairment [11]. One point was added to the total MoCA score for patients with no more than 12 years of education, as they tend to perform worse on the MoCA [12].

### Statistical analysis

Statistical analysis was performed using SPSS 22.0 (IBM, Chicago, IL, USA). Univariate simple linear regression was performed to determine the association of different variables with the total MoCA score, and variables that were significant in this analysis were entered stepwise into multivariate linear regression. Data were reported as mean ± SD, and regression coefficients were reported with a 95% confidence interval (CI). *P* < 0.05 was considered statistically significant.

## Results

A total of 30 patients with basal ganglia stroke were enrolled (Table 1). Univariate linear regression showed that the total MoCA score was significantly associated with patients’ sex, age, executive function, naming, attention, abstract generalization ability, memory ability, and visuospatial orientation. However, multivariate linear regression identified only executive function, naming, attention, memory ability, and visuospatial orientation as significantly associated with total MoCA score (Table 2).

**Table 1:** Patient characteristics (*n* = 30)

Characteristic	Value
Sex	
Male	20
Female	10
Education level	
Illiterate	3
Primary	4
Secondary	19
Tertiary	4
Etiology	
Hemorrhage	17
Infarction	13
Stroke location	
Left	11
Right	19
Age, years	58.93 ± 13.28
Montreal Cognitive Assessment scores	
Executive function	2.47 ± 1.28
Naming	2.43 ± 0.97
Attention	4.53 ± 1.50
Language function	0.53 ± 0.86
Abstract generalization	0.90 ± 0.76
Memory	2.73 ± 1.48
Visuospatial orientation	3.97 ± 1.47
Total score	17.47 ± 6.20

Values are *n* or mean ± SD

**Table 2:** Univariate and multivariate linear regression to identify variables associated with total score on the Montreal Cognitive Assessment

Variable	Univariate			Multivariate		
	*β*	95% CI	*P*	*β*	95% CI	*P*
Sex	− 0.426	− 10.027 to (− 0.973)	0.019	0.051	− 0.545 to 1.870	0.267
Education	1.691	− 1.215 to 4.598	0.243	Not included		
Etiology	− 0.033	− 5.114 to 4.314	0.863	Not included		
Stroke location	0.152	− 2.869 to 6.647	0.423	Not included		
Age	− 0.416	− 0.358 to (− 0.030)	0.022	0.041	− 0.024 to 0.062	0.362
*Montreal Cognitive Assessment scores*						
Executive function	0.837	3.029 to 5.080	< 0.001	0.320	0.952 to 2.148	< 0.001
Naming	0.796	3.582 to 6.572	< 0.001	0.117	0.005 to 1.487	0.049
Attention	0.086	2.741 to 4.363	< 0.001	0.235	0.437 to 1.501	0.001
Language function	0.223	− 1.109 to 4.326	0.235	Not included		
Abstract generalization	0.582	2.185 to 7.324	0.001	0.036	− 0.487 to 1.082	0.439
Memory	0.910	3.131 to 4.470	< 0.001	0.382	0.942 to 2.245	< 0.001
Visuospatial orientation	0.704	1.806 to 4.117	< 0.001	0.115	0.038 to 0.933	0.035

*β* standardized coefficient, *CI* confidence interval

## Discussion

Here we provide evidence that the MoCA can be used to assess cognitive impairment of patients after basal ganglia stroke, and our analysis suggests that such stroke can indeed reduce cognitive function, consistent with previous studies [13–15]. In addition, our analysis shows that such stroke can reduce function in multiple cognitive domains. Accurate assessment of early cognitive impairment after stroke and timely intervention are particularly important to achieve a high quality of life for patients [16, 17].

Frontal lobe infarction is the most common type of stroke, followed by infarction in the temporal lobe, occipital lobe, thalamus, and basal ganglia. Several studies have shown that the executive function is performed mainly in the frontal cortex, which is thought to be in the center of the lateral prefrontal cortex [18]. Consistent with this, patients with frontal lobe brain damage, especially in the left dorsal frontal lobe, have shown reduced problem-solving ability and difficulty in organizing and implementing plans [18]. The basal ganglia are a series of subcortical nerve tissues in the forebrain located below the anterior segment of the lateral ventricle. The motor region of the cortex can project into the basal ganglia through the thalamus and subthalamic nucleus, while the major output from the basal ganglia extends from the globus pallidus to the thalamus and from the nucleus to the motor cortex, the pre-motor cortical region, and the prefrontal cortex. Hence, the basal ganglia are not involved in the motor pathway extending from the cortex to the spinal cord, and therefore do not participate in the direct control of motion. Instead, they belong to the cortical–subcortical motor loop, which controls both motor and non-motor processes. Therefore, infarction of the basal ganglia can disrupt the anterior frontal lobe and subcortical loops similar to frontal lobe injury [19, 20], potentially affecting all cognitive functions. Basal ganglia stroke has been linked to impairment in executive function, but not attention deficits or delayed memory [18]. Indeed, our results support that basal ganglia stroke can reduce function in multiple cognitive domains of the MoCA. Linear regression suggested that basal ganglia stroke is closely related to impairments in executive function, naming, attention, memory ability, and visuospatial orientation.

Our study showed no significant differences in total MoCA score or MoCA subscores between patients who differed in stroke location (right vs*.* left) or etiology (cerebral hemorrhage *vs.* cerebral infarction). Similarly, we found that the total MoCA score was not associated with patients’ age, sex, or education level at the onset of basal ganglia stroke, in contrast to previous studies [21]. Our failure to detect an association between sex and cognitive subscores may be attributed to our small sample. Thus, further studies should be performed to clarify the potential influence of sex on cognitive impairment after basal ganglia stroke. Despite this limitation, our observations may help guide the development of more effective rehabilitation programs for patients with basal ganglia stroke.
